# PhyloGibbs-MP: Module Prediction and Discriminative Motif-Finding by Gibbs Sampling

**DOI:** 10.1371/journal.pcbi.1000156

**Published:** 2008-08-29

**Authors:** Rahul Siddharthan

**Affiliations:** The Institute of Mathematical Sciences, Chennai, India; Washington University, United States of America

## Abstract

PhyloGibbs, our recent Gibbs-sampling motif-finder, takes phylogeny into account in detecting binding sites for transcription factors in DNA and assigns posterior probabilities to its predictions obtained by sampling the entire configuration space. Here, in an extension called PhyloGibbs-MP, we widen the scope of the program, addressing two major problems in computational regulatory genomics. First, PhyloGibbs-MP can localise predictions to small, undetermined regions of a large input sequence, thus effectively predicting *cis*-regulatory modules (CRMs) ab initio while simultaneously predicting binding sites in those modules—tasks that are usually done by two separate programs. PhyloGibbs-MP's performance at such ab initio CRM prediction is comparable with or superior to dedicated module-prediction software that use prior knowledge of previously characterised transcription factors. Second, PhyloGibbs-MP can predict motifs that differentiate between two (or more) different groups of regulatory regions, that is, motifs that occur preferentially in one group over the others. While other “discriminative motif-finders” have been published in the literature, PhyloGibbs-MP's implementation has some unique features and flexibility. Benchmarks on synthetic and actual genomic data show that this algorithm is successful at enhancing predictions of differentiating sites and suppressing predictions of common sites and compares with or outperforms other discriminative motif-finders on actual genomic data. Additional enhancements include significant performance and speed improvements, the ability to use “informative priors” on known transcription factors, and the ability to output annotations in a format that can be visualised with the Generic Genome Browser. In stand-alone motif-finding, PhyloGibbs-MP remains competitive, outperforming PhyloGibbs-1.0 and other programs on benchmark data.

## Author Summary

Proteins in a living cell are not expressed all the time: instead, genes are turned on or off on demand. Indeed, though nearly every cell in a multicellular organism has a complete copy of the genome, each cell expresses only a fraction of the encoded proteins. Regulation of gene expression occurs in various ways. One of the most important (especially in simpler organisms) is “transcriptional regulation,” where specialised DNA-binding proteins, “transcription factors,” attach to the DNA to recruit the gene-transcriptional machinery. Detecting binding sites in DNA for these factors has long been a problem of interest in computational biology. Here, a program, PhyloGibbs-MP, is presented that extends our previously published motif-finder PhyloGibbs to handle some important related problems, in particular, detecting “discriminative” sites that distinguish differently regulated groups of genes and finding “*cis*-regulatory modules,” regions of DNA that contain large clusters of regulatory-protein-binding sites. PhyloGibbs-MP compares well on benchmarks with the best specialised programs for all these tasks, while being the first to integrate them in one consistent formalism. Regulatory regions in higher eukaryotes can be highly complex, and PhyloGibbs-MP is expected to be a very useful tool in identifying and analysing regulatory DNA.

## Introduction

Complex, carefully orchestrated cascades of gene regulatory events control various biological phenomena, from the cell cycle to stress response to the development of an organism and differentiation of its tissues. Gene regulation can be pre-transcriptional (such as by epigenetic silencing of genes), transcriptional (controlling the recruitment of the RNA polymerase), or post-transcriptional (by degrading messenger RNA before it is translated). Transcriptional regulation is mediated, in prokaryotes and eukaryotes, by specialised proteins called “transcription factors” (TFs) that bind to the DNA near a gene and recruit the RNA polymerase (or inhibit its recruitment). This regulatory control is often combinatorial, with many TFs controlling a gene, and highly complex. A gene that encodes a TF, when turned on, may cause many more genes to be turned on. To understand gene regulation, therefore, it is important to identify potentially regulatory DNA and to understand how and where individual TFs may bind there.

Typically TFs recognise short patterns or “motifs” in DNA that they bind to. For this reason, “motif-finding”, or detection of short patterns that are over-represented in a generic “background”, is an important computational problem in studying gene regulation. These motifs are generally not exact strings, but indicate weaker site-specific nucleotide preferences. Though several highly efficient substring-finding algorithms exist in computer science, they are of limited utility here. Instead, a common approach is to assume that the background is either random, or contains short-ranged correlations that can be described by a Markov model, while binding sites for transcription factors can be represented as samples from “position weight matrices” (PWMs) [1]. For a motif of length *ℓ*, a PWM is a 4×*ℓ* matrix giving the probability, at each position, of seeing each of the four bases (A, C, G, T) at that position. Two standard ways of detecting conserved regulatory sites amidst “background sequence” are Gibbs sampling, first described in this context by Lawrence et al. [2], and expectation maximisation over mixture models, implemented in the MEME algorithm of Bailey and Elkan [3].

Recently we presented a new implementation of the Gibbs sampler, PhyloGibbs [4]. The primary goal was to deal systematically with the case of orthologous sequence from closely-related species, where naive scoring of overrepresentation will fail because much sequence has not diverged sufficiently. This is handled using a user-specified phylogenetic tree and a modified scoring scheme for phylogenetically related sequence. Additionally, PhyloGibbs evaluates its own site predictions via statistical sampling of the entire state space, so that it can report the posterior probability, given all prior assumptions, that a given site is indeed a binding site. We showed that this self-assessment is an improvement on previous programs, which either do not assess their predictions, or use other statistical significance measures that do not evaluate to posterior probabilities.

A related point is that, in addition to individual site predictions, PhyloGibbs outputs weight matrices that are constructed from the predicted binding sites *weighted by their significance*, and are not merely counts of nucleotides. A problem with experimentally determined weight matrices is that they are often constructed from a small number of annotated binding sites, all of which are weighted equally, even though they may not all be of equal affinity in practice. Indeed, not all experimentally-determined sites may be known with equal confidence: binding assays often localise a much larger region of DNA, within which the putative binding sites are found bioinformatically. PhyloGibbs can be used to construct weight matrices from such data, weighted by confidence, as discussed below (Materials and Methods, “CRM Prediction”), a point that was not fully explored in the previous paper.

Here we present PhyloGibbs-MP, an extension of PhyloGibbs in several directions that go well beyond standard motif-finding. MP stands at the moment for “module prediction” (and possibly also “multiprocessor”: it has preliminary support for shared-memory multiprocessor systems, using OpenMP, and future support for distributed-memory clusters, via MPI, is planned).

- First and not least, the speed has been significantly improved (by a factor of 5 to 10) by using an “importance sampling” scheme. Further speed increases have come from improved code and more efficient data structures.
- PhyloGibbs-MP now takes account of “prior information” in the form of weight matrices for already-characterised transcription factors. This biases the search towards these known weight matrices by specifying a Bayesian prior for each site to be a binding site for each weight matrix. (A different approach to this feature, due to Erik van Nimwegen, was in the PhyloGibbs-1.0 code, though not discussed in the accompanying paper.)
- While in simple organisms like bacteria and yeast, one may safely assume that most intergenic sequence is regulatory, this ceases to be true in higher organisms. Here, one can have several tens of kilobases of potentially regulatory sequence, upstream or downstream of the gene or in introns. But binding sites for TFs are not uniformly scattered over all this sequence: they are usually localised in “*cis*-regulatory modules” (CRMs) which may be only about a kilobase or two in length. Prediction of CRMs is a long-standing research topic. Most approaches (such as Cis-Analyst [5],[6], Cluster-Buster [7], and Stubb [8]–[10]) use already-characterised PWMs to predict binding sites and then look for local “clusters” of such sites. In studying gene regulation in higher eukaryotes, predicting CRMs is a necessary first step before running a motif-finder. PhyloGibbs-MP can localise predictions to small modules, not known a priori, in large quantities of input sequence. We demonstrate that PhyloGibbs-MP in this form is a remarkably effective module predictor, which, unlike previous module-finding programs, can work a priori (without information about already characterised WMs); when fed such prior information, its effectiveness increases further. (A drawback is that Gibbs sampling is much slower than a straightforward site search for known factors.)
- PhyloGibbs-MP can also restrict the number of motifs detected *per module* to a subset of the total. For example, while it may be reasonable to assume that 20 factors regulate a set of genes totally, each CRM may have inputs from no more than 4 or 5 factors.
- Motif-finding is often improved by identifying groups of co-regulated genes, for example from microarray data, and it is common to give regulatory sequence from several genes to a motif-finder. However, one may also want to study groups of genes that are believed to be *differently* regulated (for example, microarray data analysis puts them in different clusters), and it is of enormous interest to find motifs that appear *preferentially* in one group rather than in the other. PhyloGibbs-MP now implements a “discrimniative motif-finding” option to find such motifs that distinguish each group from the others. We are unaware of any other published program that implements this feature, despite its obvious practical importance.
- Finally, for easier visualisation, PhyloGibbs-MP now outputs annotations in the Generic Genome Browser (GBrowse) format, which may be uploaded to any GBrowse server. (We are also writing a stand-alone visualisation tool to handle such data: S. Acharya and RS, in preparation).

In the Results section, we benchmark PhyloGibbs-MP in motif-finding, module prediction and discriminative motif-finding. In the Methods section, we describe the implementation of these features.

In addition, several smaller changes in the algorithm have been made, discussion of which occurs towards the end of the Methods section. Also, many command-line options are no longer compatible with the earlier program. To avoid confusion, we have renamed the program “PhyloGibbs-MP”.

In this paper, “PhyloGibbs-1.0” and “PhyloGibbs-MP” are used for statements specific to those versions of the program, and “PhyloGibbs” is used for remarks common to both programs.

## Results

### Binding Site Prediction in Yeast and Fruitfly

Before discussing new features, we first test the straightforward motif-finding capability of PhyloGibbs-MP, on test datasets of known binding sites in yeast (*Saccharomyces cerevisiae*) and fruitfly (*Drosophila melanogaster*).

### Yeast Benchmark

This is essentially a repeat of tests reported for PhyloGibbs-1.0 [4], using the SCPD database [11] of experimentally-determined transcription-factor binding sites in *S. cerevisiae*. A filtered list of these binding sites, rejecting very large and very small sites, was used; this contains 466 binding sites upstream of 200 genes. The advantage here is that every site in this database is experimentally validated; it thus provides a very good measure of real-world performance of various algorithms. The disadvantage is that there may be many sites that are not known. We previously argued [4] that we expect roughly one in three sites to be known (and present in this database), and showed that PhyloGibbs' self-assessment of its predictions is consistent with this expectation.

For each of these 200 genes, we select up to 1000 bp upstream sequence (not overlapping coding sequence) from *S. cerevisiae*, orthologous sequence from *S. paradoxus* [12], *S. mikatae*, *S. kudriavzveii*, *S. bayanus* [13], and run various motif-finders on them. The orthologous sequences were determined *ab initio* using BLAST and synteny as criteria.

The motif-finders tested were AlignACE [14],[15], MEME [3], PhyME [16], EMnEM [17], and the Gibbs sampler from the Wadsworth Institute [2]. Other than AlignACE, the other programs had been previously tested against PhyloGibbs-1.0 [4]. Here, however, we use an updated Wadsworth Gibbs sampler, which has recently acquired [18] the ability to do “phylogenetic” sampling. We tested this program both in non-phylogenetic mode and in the phylogenetic mode.

**Figure 1:**
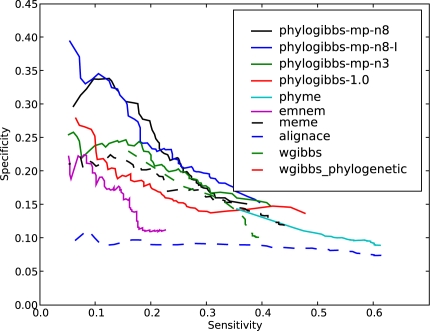
The performance of various motif-finders on predicting yeast binding-site data taken from SCPD. Specificity (the fraction of predicted sites that are present in SCPD) is plotted as a function of sensitivity (the fraction of SCPD sites that are found by the motif-finder); sensitivity is varied by cutting off predictions below a varying significance threshold as reported by the individual program. Three runs of PhyloGibbs-MP are reported: phylogibbs-mp-n8 is a run that specifies a maximum of 8 colours (types of motif); phylogibbs-mp-n8-I is the same, but with “importance sampling” turned off; and phylogibbs-mp-n3 is a run that specifies a maximum of 3 colours.

The results are shown in Figure 1, in the form of specificity (fraction of binding sites predicted that are known) as a function of sensitivity (fraction of known binding sites that are predicted).

The sensitivity and specificity are varied by choosing different “cutoff scores” for the significance scores assigned by various programs; only sites with a significance above the “cutoff score” are considered. The higher the cutoff, typically, the lower the sensitivity but the higher the specificity. In the case of AlignAce, which does not assign significance scores to individual site predictions, we used the different predicted motifs as cutoffs. (That is, the different points on the sensitivity/specificity curve correspond to the sensitivity/specificity calculated from all sites predicted in the first *n* motifs, from *n* = 1 onwards.) PhyloGibbs-1.0, PhyloGibbs-MP and EMnEM used multi-fasta sequences aligned with Sigma [19] version 1.1.3, PhyME used sequences aligned with a bundled version of Lagan [20], and the phylogenetic Gibbs sampler used sequences aligned with ClustalW [21]. All other programs used unaligned sequences.

PhyloGibbs-1.0, MEME, PhyME and EMnEM perform similarly to previously reported. AlignACE performs rather poorly on this dataset. This is probably a result of the lack of site-specific significance information in its output. The Wadsworth Gibbs sampler, run in the normal (non-phylogenetic, non-centroid) mode shows a much improved performance from the version we previously reported. However, when run in phylogenetic mode, the Wadsworth Gibbs sampler makes very few predictions indeed.

The Wadsworth Gibbs sampler in phylogenetic mode was run with a commandline suggested by W. Thompson (personal communication). Commandlines in other cases were chosen based on available documentation. Details are in Materials and Methods.

PhyloGibbs-MP is run in two modes: searching for a maximum of 3 or a maximum of 8 simultaneous motifs; and in the latter case, with or without “importance sampling”. All choices show good performance but performance is clearly superior when searching for 8 motifs, with or without importance sampling. This is in contrast to most other programs (not shown), including PhyloGibbs-1.0, where searching for too many simultaneous motifs hurts performance. Importance sampling (the default) gives a speed increase of a factor of about 10, and these data show that the effect on the quality of predictions is minor. This is further discussed in “Materials and Methods” (subsection “Importance sampling”).

PhyloGibbs-MP performs well when searching for multiple simultaneous motifs (“colours”) because it allows each colour to contain only as many windows as actually belong (that is, it enables toleration of overestimates of the number of binding sites). For example, suppose one assumes that there are 3 regulatory motifs, and 1% of a 1000 bp sequence is functional: that would yield 10 sites overall, or 3 to 4 sites per motif. In fact, however, there may be only two sites per motif. Providing more allowed colours lets the “good” motifs be grouped together, and irrelevant motifs are placed into other colours (and, since they are not selected often, do not accumulate high tracking scores). To some extent this applied to PhyloGibbs-1.0 too; but PhyloGibbs-1.0 insisted, for technical reasons, on having at least one selected site for every colour, which hurt performance when the number of colours was large.

### Fruitfly Benchmark

We used the REDfly 2.0 [22] transcription factor binding site database. Since many of these reported binding sequences are much longer than the expected length of an individual binding site, we chose a subset for which we could bioinformatically determine the likely binding site with reasonable confidence using independent data, as described in Materials and Methods. Moreover, we wanted to include PhyloCon [23], a motif-finder that makes somewhat different assumptions about the nature of input sequences (in particular, it expects multiple sets of orthologous sequences, with at least one binding site in each set). Therefore, we chose only factors for which, after our processing, multiple binding sequences were available.

Other than the addition of PhyloCon, all programs used in the yeast benchmark were run on this data, except PhyME, which we could not successfully run on this data (it crashed), and Phylogenetic Gibbs, which showed poor performance on the yeast data even after its commandline parameters were extensively adjusted on the advice of one of its authors. Also, in the PhyloGibbs family, only PhyloGibbs-MP with eight input sites was run, since this showed the best results in the yeast benchmark.

In addition to *D. melanogaster* sequence, orthologous sequence was used from the recently sequenced genomes of *D. yakuba*, *D. erecta*, and *D. simulans* [24]. (Though many papers have used *D. pseudoobscura*, the second fly genome to be sequenced, we rejected it because its distance from *melanogaster* suggests that gene evolution may have evolved significantly.)

**Figure 2:**
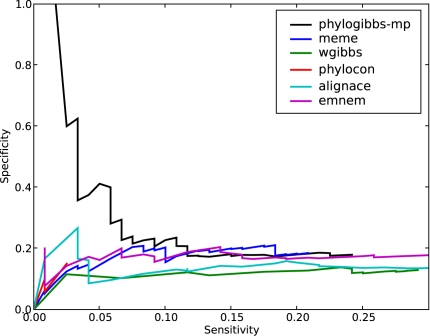
The performance of various motif-finders on predicting binding sites in *D. melanogaster* taken from REDfly 2.0. The interpretation is similar to that in Figure 1.

The results are shown in Figure 2. They are largely similar to the yeast results, but overall the sensitivity is poorer than in the yeast data: that is, all programs perform with poor specificity for a sensitivity greater than about 0.12, and their performance is probably no better than random at this level. If one focuses on high-quality predictions (which means poor sensitivity), PhyloGibbs-MP sharply outperforms all other programs. PhyloCon performs particularly poorly: a detailed look at the output shows that it predicts sites only for two factors, ftz and bcd, and most of these predictions don't correspond with the annotated sites.

It should be noted that binding motifs in fly (and other higher eukaryotes) are much fuzzier and less specific than in yeast, and (especially with the homeotic factors) tend to be rich in A's and T's; similar motifs may well occur by chance, to lead the motif-finders astray. Moreover, all the input sequences probably contained several binding sites for several factors other than the ones annotated by REDfly. Many of these unknown binding sites may have been successfully detected but not measured in the benchmark. Nonetheless, PhyloGibbs-MP performs clearly better at the high-confidence end, suggesting that it is indeed better at distinguishing conserved binding sites from background. Indeed, the top few sites predicted by PhyloGibbs-MP are all known sites (specificity 1.0) and account for about 2% of the total number of sites in this benchmark.

### Discriminative Motif-Finding

Discriminative motif-finding has been discussed in the literature previously. We choose three previously published programs, ALSE [25], Dips [26], and DEME [27], which are available for downloading and running locally, to benchmark against PhyloGibbs-MP. Other published programs, not available for download, include LearnPSSM [28], DME [29], and dPattern [30].

Unlike previous programs, PhyloGibbs-MP handles multiple input data sets, and treats them symmetrically: rather than requiring a “positive” and a “negative” set, it seeks sites in any one “discriminative group” that are over-represented in that group but under-represented in the other groups. (A set of genes A may be up-regulated relative to another set B, not only because genes in A are being activated by a common factor, but because genes in B are being repressed by a common factor. Moreover, one is often interested in the regulation of multiple sets of genes, that are differently expressed over a set of conditions, even if no one set of genes is preferentially expressed overall.) Moreover, the degree of differential discrimination may be controlled by a command-line parameter (-d). If this parameter is provided but is zero, sites are found in a single group without regard to how much they may be represented in other groups. Very high values of the parameter strongly repress prediction of sites that have counterparts in other groups (this is similar to how other discriminative motif-finders work). In addition, PhyloGibbs-MP takes systematic account of phylogenetic relationship between species.

We performed four tests: on synthetic data; on yeast data using the same SCPD database as in the previous section; on fruitfly data using REDfly binding site data, again as in the previous section; and on groups of putative co-regulated yeast genes obtained from genome-wide binding data from Harbison et al. [31]. Results from these tests suggest that making the discrimination too aggressive is counterproductive, which may account for why discriminative motif-finders have not achieved as much popularity as conventional motif-finders. On actual genomic data, the performance of PhyloGibbs-MP compares well with, or exceeds, the performance of DEME and ALSE.

### Synthetic Data Benchmark

For synthetic data, we generated two sets of phylogenetically-related sequence as follows: first, three random motifs A, B, C of 10 bp each were selected, drawn position weight matrices where the consensus base had 85% weight, with the remainder uniformly distributed. Two “ancestral” sequences were generated, each containing five copies of motif A; one of these also contained five copies of motif B, while the other had five copies of motif C. These ancestral sequences were then evolved, according to our evolutionary model with an expected mutation rate of 0.5 per nucleotide, to five descendants; mutated background bases are re-sampled from the background model, and mutated bases in binding sites are re-sampled from the PWM. Thus we have two sets of five phylogenetically related sequences, each containing 5 copies of a “common” motif and 5 copies of a “discriminative” motif. 200 such pairs of sequence sets were generated, and PhyloGibbs-MP, ALSE, DEME and Dips were benchmarked on them, for sensitivity and specificity in detecting the common motifs and the discriminative motifs.

**Figure 3:**
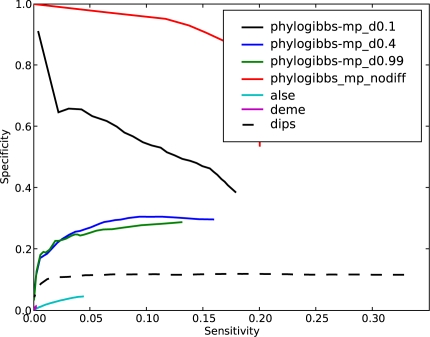
The performance of PhyloGibbs-MP in discriminative and non-discriminative mode, on synthetic data, compared with other programs. Each data set consists of two sets of sequences, with one “common” motif embedded in both sets and two “discriminative” motifs embedded one in each set, with five copies per sequence per motif per set. Specificity as a function of sensitivity is shown. For PhyloGibbs-MP, “nodiff” indicates non-discriminative mode, while the other labels indicate the value of the discriminative parameter ( -d): 0.1, 0.4 or 0.99. This figure shows performance in detecting common motifs on these data; Figure 4 shows performance in detecting discriminative motifs.

**Figure 4:**
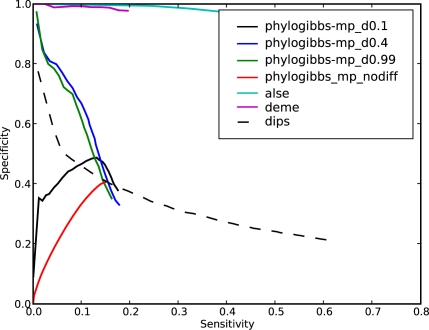
Performance of various programs in detecting discriminative motifs, on the same data as in Figure 3.

The results, with different choices of the discriminative parameter for PhyloGibbs-MP, are shown in Figures 3 and 4. When we are not seeking discriminatively occurring motifs, PhyloGibbs-MP finds the common motifs (which are abundant) with high sensitivity and high specificity, while the discriminative motifs are found with poor sensitivity and specificity. As we turn up the discriminative parameter, the significance of common motifs is reduced, and discriminative motifs are found more significantly. At “ -d 0.4”, discriminative motifs are found with moderate sensitivity but very high specificity; common motifs are significantly suppressed. Further increasing the parameter yields smaller gains.

DEME picks up hardly any common motifs, and ALSE also picks up very few. Dips picks up common motifs, but with a specificity of about 0.1 (independent of sensitivity) that indicates random performance. (Each 1000 bp sequence contains 5 embedded common motifs each 10 bp long, but overlaps of up to 5 bp in predictions would be considered “hits”; therefore about 10% of each sequence would be “hit” randomly.)

On the other hand, ALSE and DEME both perform very well in picking up differential motifs; PhyloGibbs-MP, with differential parameters -d 0.4 and -d 0.99 performs reasonably well, outperforming Dips.

### Yeast Binding-Site Data Benchmark

**Figure 5:**
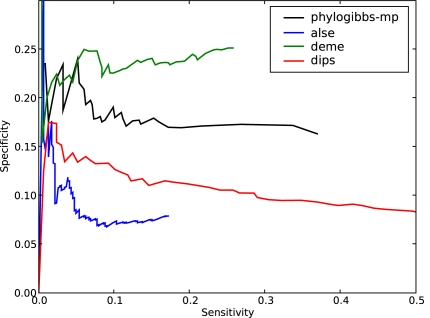
Performance of discriminative motif-finders on pairs of regulatory regions from yeast.

With yeast data, we selected pairs of genes for which differing regulatory factors are listed in SCPD, and picked 1000 bp upstream sequence (excluding overlapping coding sequence) with orthologous sequence from other *sensu stricto* species, as for the motif-finding benchmark. Having generated 571 such pairs, we ran PhyloGibbs-MP (with discriminative setting 0.4), ALSE, DEME and Dips on each pair and measured their performances. (PhyloGibbs-MP treats the members of a pair symmetrically; the other programs were run twice on each pair, alternately choosing one member as the “positive” set and the other as the “negative” set.) The results are in Figure 5. DEME is the best performing program on this set, except for high-significance (low sensitivity) predictions, where PhyloGibbs-MP is competitive with it.

In interpreting this data (and the data in the following fruitfly benchmark), one should note that, first, there could well be unknown common factors regulating many of these pairs of genes, and second, different factors may nevertheless bind to somewhat similar binding sites (since proteins have a limited number of DNA-binding domains). The second point is even more important in the fruitfly case.

### Fruitfly Data Benchmark

We used the same sequences and binding-site data as in the fruitfly motif-finding benchmark, but also included factors for which only one sequence was available. Similarly to the discriminative yeast benchmark, we chose pairs of sequence sets that contained binding sites for different *known* factors. It should be emphasised that it is very likely—even more so than in the yeast case—that these pairs of sequence sets contained unknown common binding sites, and also that many different factors in this case contain rather similar motifs.

**Figure 6:**
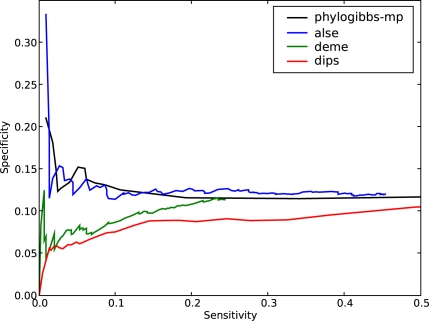
Performance of discriminative motif-finders on pairs of regulatory sequence from fly.

The results are in Figure 6. In this case, the gap between PhyloGibbs-MP and ALSE is quite low; DEME, surprisingly, performs significantly worse.

### Yeast Genomewide Binding Data Benchmark

Finally, as a test on realistic data of the sort where a discriminative motif-finder would be useful, we considered DNA-binding data from genome-wide location microarray experiments (“ChIP-chip”) reported by Harbison et al. [31] in *S. cerevisiae*. We chose 15 factors with “known” motifs (as reported by them) that bind between 4 and 9 probes, under at least two conditions including rich medium, with a *p*-value below 0.001; reported binding sequences (and orthologues) were in the “positive” set and an equal number of randomly chosen non-binding sequences were in the “negative” set. The factors thus chosen were AFT2 (YPL202C), BAS1 (YKR099W), CBF1 (YJR060W), DAL81 (YIR023W), GAL4 (YPL248C), HAP2 (YGL237C), MET4 (YNL103W), MSN4 (YKL062W), PUT3 (YKL015W), RCS1 (YGL071W), RDS1 (YCR106W), ROX1 (YPR065W), RTG3 (YBL103C), STP1 (YDR463W), YAP1 (YML007W). Details are in Materials and Methods.

**Figure 7:**
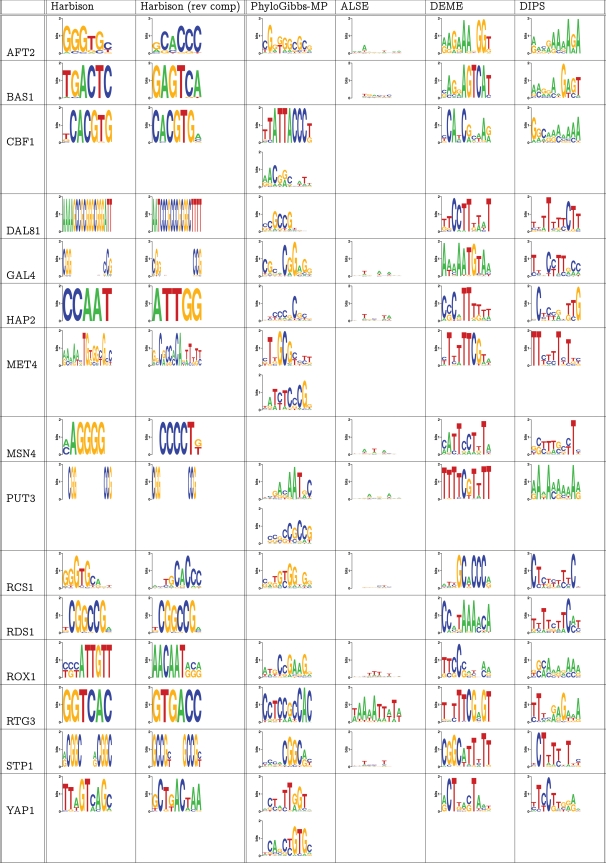
For fifteen transcription factors bound by between 4 and 9 sequences with *p*<0.001 in ChIP-chip experiments reported by Harbison et al. [31], weight matrices reported by those authors, in both orientations, compared with predictions of four discriminative motif-finders on binding sequences discriminated against randomly chosen non-binding sequences. No other prior information was used. PhyloGibbs-MP does not internally characterise discriminative sets as “positive” or “negative” but only predictions from the positive set (including, in some cases, multiple predictions) are reported. Other programs make at most one prediction per set. All programs report position weight matrices, which were used directly to generate sequence logos (using WebLogo [41] and some helper scripts). The predictions are discussed, qualitatively, in the text.

Figure 7 summarises the results. Unlike other benchmarks, the comparison here is qualitative, and at the motif-level not the site-prediction level. On the whole, most predicted motifs bear little resemblance to the “known” motifs. The various programs perform as follows:

- In 9 out of 15 predictions of PhyloGibbs-MP, there is at least a core element of the predicted motif that resembles the known motif. These are for AFT2 (GGG[T/C]GC), DAL81 (CCGC[C/G]G or CCGCC[C/G])), GAL4 (CGG), MET4 (T[G/T]GCGC), PUT3 (CCG), RCS1 (G[G/T]GTG), RTG3 (G[G/C]TCAC), STP1 (CGGC), YAP1 (TTAGT). Of these, the full GAL4, PUT3, and STP1 motifs are homodimers with weak spacers, and PhyloGibbs-MP predicts one half of these dimers. The GAL4 motif, in particular, contains a very long (11 bp) spacer which is hard to find with motif-finders, especially as the expected length of the motifs was specified as 10 in all these programs.
- ALSE predicts extremely indistinct motifs with low information content, except in the case of RTG3, where its prediction bears little resemblance to the known motif.
- DEME predicts core sequences for 8 out of 15 factors: AFT2 (GGT, much shorter than PhyloGibbs-MP's prediction), BAS1 (AGTCA, a strong prediction missed entirely by PhyloGibbs-MP), DAL81 (CTTTT), HAP2 (CCA[A/T]T), MET4 (TTTT[T/C]), RCS1 (GCACCC, a sharper prediction than made by PhyloGibbs-MP), STP1 (CGGC), YAP1 (CTGACTA, partially overlapping with PhyloGibbs-MP's prediction).
- Dips makes (comparatively weak) predictions for five factors: BAS1 (GAGT), DAL81 (TTTT), HAP2 (CCANT), MET4 (TTTTT), MSN4 (CCCT), ROX1 (AACAA).

This is a qualitative comparison and many of the above comparisons are rather nebulous. In particular, if one omits poly-A or poly-T predictions (which are plentiful in the yeast genome), the number of “predictions” for DEME and Dips falls sharply.

Though DEME makes only eight predictions that match (or somewhat match) the known motifs in these data, its authors report benchmarks that predict 13 of 15 motifs in sequences drawn from the same ChIP-chip data [27]. However, both their selection of sequences (which is not described in detail) and their methodology differ from ours. In particular, where we assume no prior knowledge of the motif width and use a width of 10 in all cases, they use the actual width of the motif as prior information. In realistic situations, one is unlikely to know the width of an uncharacterised transcription factor's binding site.

### Interpretation of Discriminative Benchmarks

When one takes the four benchmarks together—one on synthetic data and three on actual genomic data of the type likely to arise in realistic research situations—it is clear that, in this particular problem, synthetic data captures very poorly the complexities of an actual situation. There are various issues at work in actual genomic sequence: motifs for different transcription factors may resemble each other strongly, especially if they come from the same family; weak, non-specific motifs may have close matches by “chance”; there could be many relevant factors regulating a gene or set of genes, only some of which are discriminative; and, in data arising from high-throughput experiments, there could be “noise” in that not all sequences reported to bind a protein may actually do so. Over-aggressiveness in discriminative motif-finding leads to excellent synthetic-data performance but poor sensitivity and/or specificity with real genomic data. All the programs tested suffer from this issue, but PhyloGibbs-MP mitigates the problem with the tunability of its discriminative parameter.

The benchmarks do indicate that, over a somewhat broad range of data, DEME and ALSE are excellent discriminative motif-finders, performing far better than PhyloGibbs-MP on synthetic data and very well on real data. However, with real data, PhyloGibbs-MP is competitive with or superior to both those programs (in particular, it appears markedly superior on the ChIP-chip benchmark), while including the flexibility of a general-purpose motif-finder.

### CRM Prediction

**Figure 8:**
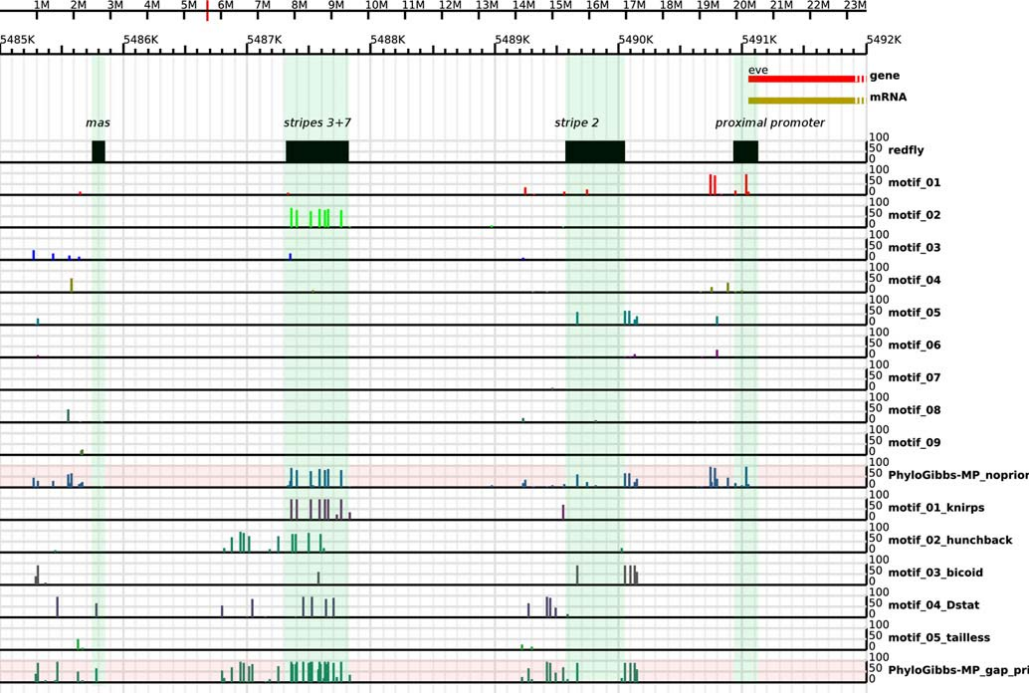
Results of running PhyloGibbs-MP, in module-prediction mode, on the 8 kb sequence upstream of the *eve* gene in Drosophila. When run without priors, predictions lie on or close to all four annotated modules in this region from the REDfly database. When weight matrices for the gap transcription factors are used as priors, PhyloGibbs-MP fails to find the proximal promoter, but the stripe 2 and stripes 3+7 enhancers are detected with increased confidence. Predicted sites for individual motifs, as well as cumulative predictions over all motifs, are shown.

An example of PhyloGibbs-MP's ability to predict *cis*-regulatory modules is shown in Figure 8, which depicts the region upstream of the *eve* gene. Without prior information, PhyloGibbs-MP successfully predicts all four annotated upstream CRMs from the REDfly database: the proximal promoter, the stripe 2 enhancer, the stripes 3+7 enhancer, and the mas enhancer. (In the case of mas, the predictions are not exactly overlapping the annotated enhancer, but are nearby and over a broader region. In the case of stripe 2, the annotated 500 bp enhancer in REDfly is somewhat shorter than other reports (e.g., Ludwig et al. [32]) that suggest an enhancer close to 800 bp in length; most of the predicted sites fall in this larger region. With prior information in the form of nine weight matrices corresponding to gap factors, PhyloGibbs-MP fails to pick up the proximal promoter, but finds the remaining CRMs with greater confidence than before.

For a more thorough and quantitative benchmark, we used the REDfly database [22],[33] of *cis*-regulatory modules in *D. melanogaster*. We filtered the REDfly CRM list for CRMs of suitable length (<3000 bp), fused nearby CRMs, and selected sufficient neighbouring sequence (details are in Materials and Methods) that we were left with 234 stretches of DNA that were at least 10000 bp long and contained at least one annotated CRM.

PhyloGibbs-MP, and four other downloadable programs—Stubb [8],[9], Cluster-Buster [7], EMCMODULE [34], and CisModule [35]—were run on these segments. (Details and exact command-line parameters are in Materials and Methods.) Cis-Analyst [36],[37], and other programs that are not downloadable or can only be run via a web-server, were not tested. For priors we used a set of 73 weight matrices, that we created from DNAse I footprints in the FlyReg database [38] and orthologous sequence in five other species (details of how these matrices were generated are in Materials and Methods, and the matrices themselves are available at http://www.imsc.res.in/∼rsidd/phylogibbs-mp/supporting-data/.

As in the motif-finding case, sensitivity was varied by varying the significance cut-off of individual site predictions (or, in the case of Stubb, of individual free-energy “windows” of 100 bp each). The sensitivity of the output was measured, for predictions above various cutoff thresholds, by what fraction of CRMs (weighted by length), of the total known, were successfully predicted by the programs. A predicted site that lay within the boundaries of a module was counted as a “prediction” of that module. The specificity was measured by what fraction of site predictions, for each program, occurred within known CRMs.

**Figure 9:**
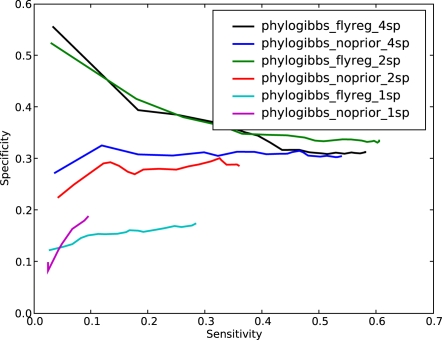
Performance of PhyloGibbs-MP with various parameter settings (with flyreg priors or without priors, and with 1, 2 or 4 orthologous aligned sequences), on detecting known *cis*-regulatory modules in regulatory regions of fly.

To test the effect of prior information and orthologous sequence availablity on the performance of PhyloGibbs-MP, it was run with and without priors, and with one (*D. melanogaster* only), two *D. melanogaster* and *D. yakuba*) or four (*D. melanogaster*, *D. yakuba*, *D. simulans* and *D. erecta*) aligned species. The results are in Figure 9. The best performance came when prior information was supplied, but (somewhat surprisingly) when only two input species, not four, were used. This suggests that spurious conservation across multiple species may lead PhyloGibbs-MP astray.

**Figure 10:**
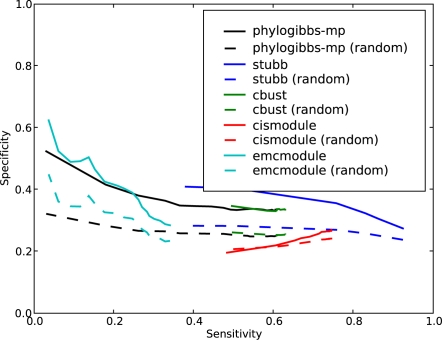
Performance of PhyloGibbs-MP (with flyreg priors, and 2 species) in detecting known CRMs in fly, compared with four other module finders. Dotted lines indicate the performance expected if programs made predictions at random (that is, if, for each input sequence, the same number of site predictions were made but at random locations). Note that, in this data, 816457 bp out of 2448515 bp is in annotated CRMs; so a completely random program would exhibit roughly a specificity of 0.33, in agreement with the dotted lines at high sensitivity.

Next, PhyloGibbs-MP (best-performing parameters) was compared with the output of the other programs. The results are in Figure 10.

In terms of speed, PhyloGibbs-MP is much slower than the other programs: being a Gibbs-sampling ab initio motif-finder, it runs roughly quadratically in sequence length (given typical parameters), whereas a search and clustering for motifs can be done in linear time. Its utility becomes apparent when used as a combined CRM predictor and ab initio motif-finder, and it performs competitively with dedicated programs at both these tasks. In recent work, we have used a subset of the above FlyReg-derived matrices, and a few additional literature-derived weight matrices, to make detailed studies of a myoblast-related enhancer that exhibits a complex modular expression pattern (K. G. Guruharsha et al., in preparation).

## Discussion

While transcriptional regulation, especially in most eukaryotes, is only one component of a complex machinery controlling gene expression, it is an important and the best understood component today. According to benchmarks on two different species reported here, PhyloGibbs-MP appears to be superior to all other tested programs (including its predecessor PhyloGibbs-1.0) in its core task of ab initio motif finding. In addition, it shows excellent performance, comparable or superior to that of dedicated programs, at prediction of *cis*-regulatory modules (CRMs) and discriminative motif-finding. All these are of importance in the computational study of transcriptional gene regulation. While comparably good or faster tools may exist for module prediction and discriminative motif prediction, the ability to integrate all these tasks, while taking systematic account of phylogenetic relationships between species and performing a careful self-assessment of its own predictions via extended sampling, are unique to PhyloGibbs-MP.

There are some subtleties and many unaddressed problems that we intend to address in future; we discuss some of these below.

While PhyloGibbs-MP predicts multiple CRMs upstream of a single gene, and regulatory sites that occur differentially upstream of different genes (as well as CRMs known a priori for the same gene), it is incapable of predicting discriminative sites for different CRMs that it itself predicts for a given gene. Yet the reason that a gene may have multiple CRMs is precisely that it is differently regulated by different CRMs in different contexts. At the moment, we approach this situation by first predicting CRMs, using either PhyloGibbs-MP or another program, and then predicting discriminative motifs in those CRMs. Also, PhyloGibbs-MP can restrict the number of different factors (colours) per CRM: for example, we can stipulate that there may be up to 40 factors regulating the gene, but only 10 at most per CRM. A more satisfactory solution will be implemented in the future.

Co-regulated and differentially-regulated clusters of genes are often predicted from high-throughput (microarray) expression data, which is itself noisy. Feedback between motif-finding and clustering of microarray data would benefit both tasks, and this is a future goal.

PhyloGibbs-MP, like its predecessor and like other phylogenetic motif finders, requires pre-aligned input data; we have written a program, Sigma, specifically for aligning non-coding DNA, and use it in the benchmarks above and elsewhere. But no alignment program is perfect, so it is a goal to include the capability of aligning sequence (using an algorithm similar to that implemented by Sigma) in PhyloGibbs-MP directly. The alignment can then be refined in tandem with the predictions being made by PhyloGibbs-MP.

### Availability

PhyloGibbs-MP is available at http://www.imsc.res.in/∼rsidd/phylogibbs-mp/. It is open source, licensed under the GNU General Public License. Supporting data is available at http://www.imsc.res.in/∼rsidd/phylogibbs-mp/supporting-data/.

## Materials and Methods

### Overview of the PhyloGibbs Algorithm

The PhyloGibbs algorithm was described in detail earlier [4], so a brief summary will suffice here. (Some changes in PhyloGibbs-MP from PhyloGibbs-1.0 are described in Materials and Methods, “Changes to Algorithm”.)

PhyloGibbs models “generic” non-coding DNA sequence by a Markov model of order *k* (where typically *k* is 1, 2, or 3) whose parameters are estimated (preferably) from an auxiliary file of background sequence, or (less reliably) from the input sequence itself. It assumes that some locations in the input sequence are binding sites for transcription factors, and are not described by the background model but by “position weight matrices” (PWMs): matrices of order 4×*ℓ* that give the probabilities of seeing each of the four nucleotides at positions 1 through *ℓ* in the site. All binding sites belonging to a common transcription factor are given by the same (often unknown) PWM. A “parse” of the sequence consists of a selection of particular sites as putative binding sites. For each such parse, *C* the likelihood of seeing the given sequence *P*(*S*|*C*) can be calculated (as described in [4]), and then the posterior probability of *C* follows by Bayes' theorem:
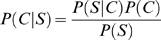
(1)Here, *P*(*S*) (loosely, the “prior probability” of the sequence *S*) is a constant, equal to Σ*_C_P*(*S*|*C*′)*P*(*C*′), while *P*(*C*) is the prior probability of the parse, which is chosen to incorporate as much prior information as possible. For example, it can be chosen to be constant for a given number of colours and a given number of sites, and zero otherwise. One can also use a “chemical potential” to allow flexibility in the number of allowed sites. PhyloGibbs-MP fixes a *maximum* number of colours, and an expected number of binding *sites* (in contrast, PhyloGibbs-1.0 fixes the number of windows, that could encompass many sites). PhyloGibbs-MP also adjusts *P*(*C*) when dealing with informative priors, module prediction, and discriminative motif-finding.

PhyloGibbs uses a moveset that preserves detailed balance (some caveats apply to PhyloGibbs-MP: see the appendix). It samples the space of parses, first finding the parse with maximum a posteriori probability *P*(*C*|*S*), then evaluating the significance of each predicted site by further sampling. It can simultaneously detect binding sites for multiple different TFs by labelling each with a different “colour”; the posterior probability is a product over all colours.

In addition, PhyloGibbs can deal with phylogenetically related sequence that has been pre-processed by a multiple sequence alignment program. It does this by treating sites in an aligned block not as independent, but as descendants of a common ancestor, and modifying the scoring appropriately (whether as “binding sites” or as “background”). The scoring is governed by the transition probability
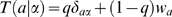
where *α* is the (unknown) ancestral base, *a* is the descendant base, *q* is the rate of conservation (“proximity”) between the ancestor and the descendant, and *w_a_* is the probability of seeing *a* at that position in the descendant under the assumption that this is a binding site for a weight matrix (or a background site, as the case may be). In other words, the ancestral site is conserved with probability *q* and mutated with probability 1−*q*; if mutated, it has undergone fixation that preserves the functionality, or the background statistics. This transition probability is transitive and has the correct limits at extreme *q*. The above applies directly to “star phylogenies” (where all species are independently descended from a common ancestor), but arbitrary phylogenetic trees are handled by converting them into sums of products of “subtrees” that individually have “star phylogenies”. (This is exact, but approximations are made in dealing with the subtrees.) Internally, PhyloGibbs represents such related sites by “windows”, aligned blocks of sequence that are either all functional, or all non-functional. Details, again, are in the earlier PhyloGibbs paper [4] and are unchanged in PhyloGibbs-MP.

### *Cis-*Regulatory Module Prediction

PhyloGibbs-MP takes as optional parameters the maximum length *l* of a *cis*-regulatory module, and the average spacing *d* between two CRMs. Then, on each input sequence, it requires that not more than max(1,(*L*+*d*)/(*l*+*d*) modules exist, where *L* is the length of the sequence.

Each site, or multispecies “window”, that is selected now must satisfy existing module constraints on all sequences that it is a part of. In other words, only windows that can satisfy the current constraints are sampled for. For example, if two modules are allowed on a sequence, but all windows that currently occupy that sequence fit within the width of one module (any two windows are within a distance *l* of each other), a new window can be sampled anywhere on that sequence (provided it satisfies constraints on other sequences that it is a part of), and will define a new module. However, if the currently selected windows must be spread across two modules, newly selected windows must fit within the constraints of those two modules—that is, they must be no more than *l* nucleotides away from any window in their module.

Thus, the allowable window placements, and the modules defined by the selected windows, are dynamically updated and need not stay localised as sampling proceeds. At the end of the run, the tracking scores, visualisable via the GBrowse annotation (see Figure 8) for an example), reveal the positions of the predicted modules, which may be sharply localised or may be spread all over the sequence.

### Discriminative Motif-Finding

When given sets of regulatory regions for genes that are believed to be regulated differently, it is of interest to find motifs that occur preferentially in one set rather than in the other. When run in discriminative mode, PhyloGibbs-MP accomplishes this by, for each “colour”, selecting sites only in one regulatory set. However, it also selects “mirror” sites in other groups. The total number of mirror sites, across all groups and all types of motifs (“colours”), is the same as the total number of actual binding sites expected.

The mirror windows are sampled for in the same way as the “real” windows, by scoring them together with the real windows. Thus, if a colour has *n* real windows and *m* mirror windows selected, a new window is sampled with the posterior probability *P_n_*_,*m*_ of being drawn from the same weight matrix as these *n*+*m* sites. It is possible for a colour to contain no mirror windows. If a colour gets emptied of real windows, the mirror windows are also emptied and re-sampled into other colours.

These “posterior probabilities” are calculated as described in our earlier paper [4]. However, it is convenient below to use the language of thermodynamics. By analogy with the Boltzmann probability of finding a thermodynamic system in a state of free energy *F*, which is equal to exp(−*βF*) where *β* is the inverse temperature, we define a “free energy” 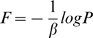 corresponding to a posterior probability *P*. In our case the temperature is fictitious: *β* = 1 during the initial equilibriation and tracking phases, and is slowly increased during the simulated anneal.

In the usual (non-discriminative) Gibbs sampler, we start from a state with *n*+1 windows, remove a window (to leave *n* windows selected), and sample replacements according to the “free energies” *F_n_*_+1_, which correspond to the posterior probabilities that the *n* selected windows plus the one new window are all sampled from the same position weight matrix. (More accurately, the new window may be placed into any existing “colour”, not necessarily the one from which a window was removed. Below we write a “colour index” explicitly for clarity.)

For the discriminative motif-finder, in any given configuration, each colour has “real” windows as well as “mirror” windows selected. A move starts with removing a real window, and a mirror move by removing a mirror window; these are then resampled. Let's say, after the removal, one is left with *n* real windows and *m* mirror windows. The “mirror” sites are sampled as above, but treating real and mirror sites as the “same”. That is, they are sampled using the free energies *F_n_*_+*m*+1,*c*_, which correspond to the posterior probability of all *n* real windows, all *m* mirror windows, and the newly selected mirror window all being sampled from the same PWM (that corresponds to colour *c*). The goal here is to maintain a mirror list that is as faithful as possible to the real list.

The “real” sites are instead sampled according to
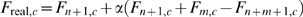
(2)where *c* stands for the colour (motif type) into which the window is being sampled *F_n_*_+1_ has the same meaning as in the non-discriminative case, *F_m_* is the free energy for the case that the *m mirror* windows are sampled from a common PWM (not necessarily the same as the “real” windows), and *F_n_*_+*m*+1_ is the free energy for all *n* selected windows, all *m* mirror windows and the one newly selected window are *all* sampled from the same PWM. A window may be sampled into any colour, and each colour has a different set of mirror windows associated with it (thus *n* and *m* both depend on *c*); so this free energy is calculated for each candidate window and for each colour into wich it may be sampled. The bracketed term has the effect of penalising cases where the mirror windows strongly resemble the real windows. Here, *α* is a parameter, greater than 0, that determines how strongly to penalise motifs that are well represented outside the current discriminative group. For very small *α*, sites for a given colour are selected only from one discriminative group but little consideration is made to whether similar sites occur elsewhere. For larger *α*, similar sites in other discriminative groups will penalise the score more severely. Typically we find that *α* = 0.4 suffices to predict genuinely discriminative motifs, while excessively large values of *α* will cause chance occurrences of the motif in other groups to excessively penalise the “good” motifs—which is also noticed in the benchmarks for discriminative motif-finders (cf. section “Results”, subsection “Discriminative motif-finding”).

### Informative Priors

Optionally, PhyloGibbs-MP can take a file containing prior position weight matrices corresponding to possibly relevant transcription factors, and bias its search to sites corresponding to those prior PWMs. This is done as follows: Input sequence is pre-parsed in PhyloGibbs-MP (as in PhyloGibbs-1.0), into “windows” of specified length. When prior PWMs are given, each prior PWM is associated with a unique “colour”. Then each window is given a prior probability of being a binding site for each of the given prior PWMs, as well as of being background. This probability is calculated as follows: Let *P*_bg_ be the probability that the window is background (estimated from a background model), and *P_δ_*(*W*) be the probability that the window contains a binding site for PWM *W* with offset *δ* from the start of the window. Then the probability that the window is a binding site for *W* is given by
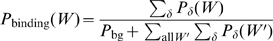
(3)where only offsets where at least 50% of the PWM is in the window, or at least 50% of the window is covered by the PWM, are considered in the sums over *δ*. (PWMs that are too large or too small to satisfy these criteria are filtered out.) Then, during the window-shift moves, when a new window and colour are being sampled, the posterior probability calculated for the new configuration is multiplied by the prior probability of that window being in that colour.

### Annotations

Optionally, PhyloGibbs-MP can output annotation files for its predictions that are readable by the Generic Genome Browser [39] as well as by a visualisation tool that we are developing and have used to generate Figure 8 in this paper (S Acharya and RS, in preparation). Annotations are for one species only, the “anchor species” that must be the first specified in the phylogenetic tree. The headers for sequences from that species must be formatted appropriately; details are in the PhyloGibbs-MP manual, distributed with the software.

### Changes to Algorithm in PhyloGibbs-MP

While PhyloGibbs-MP can be used as a conventional motif-finder, for the most part in the same way that PhyloGibbs-1.0 can, several changes have been made to the details of the algorithm, with an aim at improving performance.

### Detailed Balance

PhyloGibbs-1.0 strictly maintained detailed balance in all movesets; PhyloGibbs-MP is not quite so rigid. We discuss the deviations below. While we would prefer to maintain detailed balance strictly, we note that detailed balance, combined with ergodicity, ensures a sampling of state space with the appropriate posterior probability distribution only in the infinite time limit. But a useful program must run in limited time, and therefore good convergence of the moveset is equally important in practice. (For example, in PhyloGibbs-1.0, the “colour-change move” [4] is by itself ergodic and satisfies detailed balance, and therefore should suffice in the infinite time limit. But in any realistic running time it does nothing useful by itself. Similarly, in the infinite-time limit the “global shift moves” would not be necessary.)

We argue that our breaking of detailed balance is sufficiently rare to be harmless, and sufficiently useful to be justifiable. Rare detailed-balance-breaking moves correspond to having several Markov chains through state space, where links within a chain satisfy detailed balance, but the moves connecting different chains do not. If the number of links within a chain is much greater than the number of chains, (in particular, if each chain is allowed to grow infinitely long while the number of different chains remains finite), the desired posterior probability distribution will be reached (since it is reached separately by each of the chains).

### Window-Shift Moves

In PhyloGibbs-1.0, these moves maintained a constant number of *windows* (which may contain multiple orthologous sites), by replacing one window with another. In PhyloGibbs-MP, the window-shift move maintains a constant number of *sites*.

This is done as follows: First, an initial input parameter is *p* (by default 0.01), which is the expected “density” of sites in the input sequence. For example, if the input sequences have lengths *L_i_*, the total expected number of sites in these sequences is *N_E_* = Σ*_i_p*(*L_i_*−*w*+1) where *w* is the width of a window.

Each window-shift move removes an existing coloured window (randomly chosen, of any colour), and replaces it with a new coloured window. However, the weight (posterior probability) of the move is multiplied by the heuristic exp(−*β*((*N_s_*−*N_E_*)/*N_m_*)^8^). Here, *N_s_* is the number of sites that would be selected if that window were picked, and *N_m_* is the maximum number of sequences in any window in the set, and serves as a “margin” for the amount that *N_s_* can deviate from *N_E_*. The inverse temperature *β* is unity except during the simulated anneal, where it is increased gradually. We use the eighth power to allow deviation with little penalty within the margin *N_m_*, but rapidly growing penalties for any larger deviation.

This satisfies detailed balance. However, we make two exceptions: if, before the move, *N_s_* (the number of selected sites) is smaller than *N_E_*−*N_m_*, we do not remove any window, but directly pick a new window. And if, after removing a window, *N_s_* is larger than *N_E_*+*N_m_*, we do not pick a new window. These exceptions occur sufficiently rarely that the breaking of detailed balance is not serious. The ability to specify a “density of sites”, independent of the input sequence length or the degree of homology, is a large advantage, which contributes to PhyloGibbs-MP's superior performance over PhyloGibbs-1.0.

Another breakage in detailed balance occurs when module prediction is enabled: adding or removing windows may change module boundaries, with the result that the set of available states is different before and after the move. Again, this is relatively rare and in a useful cause.

### Global-Shift Moves

In this move, an attempt is made to shift all windows of a given colour by a fixed distance, left on one strand or right on the other. This is to move out of “local minima” where the sampler has found a non-optimal solution that is offset by a fixed amount, and moving one window at a time would take a long time to happen. To maintain detailed balance, PhyloGibbs-1.0 sampled all possible shifts, up until such distance as no shift was possible (for instance, because it was blocked by other windows, or because it would run off the edge of the sequence). While this does maintain detailed balance, it fails to sample some legitimate shifts: in particular, if a window was at the edge of an aligned block, PhyloGibbs-1.0 could not shift it beyond, because the number of sequences in the window would change, and therefore would not shift any window in that colour.

Instead, PhyloGibbs-MP samples shifts of only one space left or right, and allows shifts beyond window boundaries (such windows may either “gain” sequences from other species, or be broken into smaller windows with fewer sequences).

In this case, general balance is significantly broken for a given global shift move, because the set of available states is not the same before and after the move. (A window at position *i* could be shifted, with other windows of that colour, to *i*+1 or *i*−1 or could stay at *i*; if it shifts to *i*+1, the available states are now *i*, *i*+1, *i*+2. Also, in case of blocked windows that are “thrown away” after the shift, new windows are resampled, and again the set of possible states is not the same.) Again, the rareness of the moves compared to the window-shift moves, and their utility in practice, justifies the breakage.

### Colour-Change Moves

The colour-change move in PhyloGibbs-1.0 has been removed. Instead, a maximum number *N_c_* of colours is specified, but (unlike in PhyloGibbs-1.0) fewer than *N_c_* colours may actually be selected at any time.

### Mask Bits

PhyloGibbs-1.0 had an optional “maskbit flip” move, where certain columns are optionally not scored; these columns are sampled for using Metropolis moves. In practice, however, motifs tend to be strong at the centre and weak at the edge, except for symmetric dimer motifs (mainly in bacteria), where they can be weak in the middle. Therefore PhyloGibbs-MP allows such unscored columns only at the edge of the window (and, in the case of symmetric motifs, in a contiguous symmetric block at the centre). The advantage of this is that, when a length *w* is specified, smaller motifs (for example, of length *w*−1 and *w*−2) may also be found.

### Simulated Anneal

PhyloGibbs-1.0 used an annealing schedule where *β* (the inverse temperature) was increased linearly from 1.0 to a final lower value, followed by a short “deep quench” with *β* = 20.

Instead, PhyloGibbs-MP uses the “free energy” *E* (the logarithm of the posterior probability), averaged over a cycle, to determine the start and stop of the simulated anneal. The starting temperature is chosen to be a value where the fluctuations in energy, 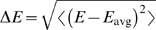 (averaged over one cycle of moves), are at least 0.3 times the average energy *E*_avg_. Then *β* is increased exponentially, by a factor of 1.2 at each step. At least two cycles are run at each *β*, and *β* is increased only when the difference in average energy in the last two cycles at that *β* is less than the fluctuation Δ*E* in the last cycle. The anneal is stopped when the relative difference in average energy at the last two values of *β* is less than 0.005. There is no “deep quench”.

The number of moves used in the tracking phase is, by default, the same as the number (excluding the equilibriation moves to find the initial temperature) in the simulated anneal. This can be overruled.

### Importance Sampling

A significant improvement in running time (typically a factor of 10 or so) is obtained by using a form of “importance sampling” on top of the Gibbs sampling scheme implemented in the “window shift moves”. When sampling a replacement window, PhyloGibbs-1.0 would consider *every* available window, with every possible new colour for that window; this requires *N_w_N_c_* calculations, where *N_w_* is the number of windows and *N_c_* is the number of colours available. Typically *N_w_* is large, several thousands or tens of thousands; but only a small fraction of available windows tends to get selected. This is not an unusual situation in sampling problems. When one has an estimate of the bias, one often uses an “importance function” *F*(*C*) to indicate which configurations *C* are more frequently visited. This is chosen (often heuristically) to be large where *P*(*C*) (the posterior probability of *C*) is likely to be large large, and small where *P*(*C*) is small. Then states are sampled not according to *P*(*C*) but according to *P*(*C*)*F*(*C*); but their contribution to running averages of the form 〈*E*(*C*)〉*_C_* are taken to be not *E*(*C*) but *E*(*C*)/*F*(*C*). This makes the average come out right, while causing the sampler to spend most of its time in “important” parts of configuration space.

In our case, we have a related situation: some windows tend to be selected much more often than others, and therefore, during a particular Gibbs move, although we need to select from *N_w_* windows, only a few of these are actually likely to be selected. Therefore we maintain an “importance” for each window, a number between 0 and 1, which is the fraction of time that that window has actually been selected (in any colour and either orientation) up until that time. During the setting of the initial temperature, all windows are treated as important (their importance counter is incremented whether or not they are selected). Thus, at the start of the anneal, all windows have importance 1, but as the running time proceeds, the importance of many windows decreases rapidly.

Let the importance of a window at any time be *I*(*w*). Normally, if *N_w_* windows may possibly be selected, we need to calculate the posterior probability *P*(*w_i_*,*c*) for each available window *w_i_* into each available colour *c*. The time-consuming step is the calculation of *P*(*w_i_*,*c*) which is a wasted calculation for most available windows, which are in fact never selected. Therefore, we pre-select a subset of the *N_w_* windows: Each available window *w_i_* is selected to be sampled with a probability proportional to *I*(*w_i_*). (*I* is normalised in such a way that the importance of the most important available window is 1, i.e. it will always be pre-selected, so the pre-selected subset is never null.) However, it will then be selected with a probability proportional, not to *P*(*w_i_*,*c*), but to *P*(*w_i_*,*c*)/*I*(*w_i_*). In other words, if a subset {*W*} of windows is preselected by importance, windows in that subset are sampled according to
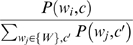
(the importance cancels because the *P*'s have been preselected with probability *i*, but then been multiplied by 1/*I*).

When the reverse transition is considered, in individual cases a *different* subset of windows {*W*′} will be used in the denominator, and therefore the sum in the denominator will not be the same. However, *on average* (for times not too far apart) it will be the same: there is no systematic bias and detailed balance should apply. In the long time limit, *I*(*w_i_*) will tend to
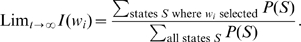
(4)This is a non-rigorous argument, for which the justification is as earlier: statements about detailed balance are rigorous only in the infinite-time limit, while running-time efficiency is important in real life. For those who are unconvinced, importance sampling may be turned off by a command-line parameter. Results do not seem greatly affected by this. An example is in Figure 1, where PhyloGibbs-MP with 8 possible motifs is run with (black line) and without (blue line) importance sampling. The effect of importance sampling seems, as one would expect, to somewhat inflate the reported significance of high-confidence predictions and somewhat deflate the significance of lower-confidence predictions.

### Benchmarks

For all benchmarks, detailed commandlines for individual programs and all input and output files are available at http://www.imsc.res.in/∼rsidd/phylogibbs-mp/supporting-data/. Several scripts used to process the data are also included.

### Motif-Finding

For the yeast benchmark, the SCPD database [11] was used, but edited to remove very long and very short sites. The edited database contained 466 binding sites upstream of 200 genes. For each gene, sequence upstream of that gene in *S. cerevisiae* up until the next coding sequence was used, to a maximum of 1000 bp. Orthologous sequence was found in other *sensu stricto* species (*S. bayanus*, *S. mikatae*, *S. kudriavzveii*, *S. paradoxus*) using BLAST.

For PhyloGibbs-1.0, PhyloGibbs-MP, and EMnEM, these sequences were aligned with Sigma [19], version 1.1.3. For the phylogenetic Gibbs sampler [18], the sequences were aligned with ClustalW [21], and for PhyME [16], they were aligned with the bundled version of Lagan [20].

Command-line options were chosen to be roughly comparable for all programs; for the phylogenetic Gibbs sampler, they were suggested by one of the authors (W. Thompson, private correspondence).

For the fly benchmark, we used the REDfly 2.0 [22] database of transcription factor binding sites. The “sites” in this database are often many times longer than the expected length of an individual binding site, so the most probable binding site needed to be found bioinformatically. Therefore, we selected a subset of these binding sequences corresponding to factors for which high-quality position weight matrices were available from the TRANSFAC 7.0 public database [40], and used these matrices to find the most probable (highest log-odds) binding sites within the binding sequences in the database. (As noted in “Module prediction”, we have independently constructed position weight matrices for a much larger set of factors from the Flyreg [38] database, which formed the basis for REDfly's binding site database, using PhyloGibbs-MP. But for the purposes of this benchmark, we prefer to use matrices of a “neutral” origin.)

The specific factors chosen from the TRANSFAC database were Abd-B, Adf1, Cf2, Dfd, Eip74EF, Stat92E, Su(H), Ubx, bcd, dl, ftz, hb, ovo, pan, sna, z. Binding sites located less than 200 bp apart were clubbed together; then surrounding sequence was selected, such that the total length of the sequence was 250 bp per binding site, to a maximum of 2000 bp.

In addition to the programs benchmarked on the yeast data, we wanted to include PhyloCon [23], a somewhat different category of motif-finder that requires multiple input sequences, each with its own orthologous sequences, and expects one site per input sequence. We therefore chose only a subset of the above factors for which, after the above process, multiple binding sequences were available for each factor. These were Adf1, Cf2, Stat92E, Su(H), Ubx, bcd, dl, ftz, hb, ovo, and sna.

Orthologous sequence was identified in *D. yakuba*, *D. erecta* and *D. simulans* using multiple sequence alignments from VN Iyer, DA Pollard and MB Eisen (personal communication), but were re-aligned using Sigma.

### Discriminative Motif-Finding

For the synthetic-sequence benchmark, 200 sets of input files were generated. Each set consisted of two files; each file contained five sequences. These sequences were generated from a single ancestor, containing five copies each of two embedded motifs, and evolved according to the evolutionary model assumed by PhyloGibbs, with *q* = 0.5 and no indels. (As in the evolutionary model, embedded sites, when mutated, were re-sampled from the weight matrix that described them.) Three motifs were used for each set, where one was common to both files in the set and one each was unique to each file in the set. The motifs were random weight matrices with a “polarisation” (maximum element in each column) of 0.85 (with the remaining weights 0.05 each). The performance of PhyloGibbs-MP (with different settings of the “discriminative parameter” -d), ALSE, DEME and Dips in detecting the common, and the discriminative, motifs was measured.

For the yeast motif-finding benchmarks, 571 pairs of genes were chosen such that the pairs had no documented binding sites in SCPD from a common transcription factor. As in the motif-finding case, orthologous sequence from the four other *sensu stricto* species was used. PhyloGibbs-MP was run in discriminative mode (-d 0.4), and performance in detecting known sites was compared with ALSE, DEME and Dips.

For the fly benchmarks, we used the REDfly data used in the motif-finding example, but removed the PhyloCon-imposed requirement of multiple sequence sets per transcription factor (allowing both single and multiple sequences per set). We ended with 1404 pairs of sequence sets in which each member of a pair was associated with a different factor. As in the motif-finding benchmark, orthologous sequence from *D. yakuba*, *D. erecta*, and *D. simulans* was used. Similarly to the yeast case, the four discriminative programs were run.

For the yeast ChIP-chip benchmarks, we used the spreadsheet Harbison_Gordon_yeast_v9.11.csv from the supplementary data of Harbison et al. [31] to extract transcription factors that bind to between 4 and 9 regulatory sequences, both in rich medium and in at least one other environmental condition, with a *p*-value better than 0.001; and for each factor we retrieved the sequences it bound to. We used intergenic sequence upstream of the regulated gene, to a maximum of 1000 bp, as in the SCPD benchmarks. 17 such factors were found, of which 15 included motifs reported in their supplementary data file Final_InTableS2_v24.motifs. Figure 7 lists these factors, and sequence logos constructed from the motifs listed in that file.

For each factor, also, if it was reported to bind to *n* sequences (4≤*n*≤9), we selected *n* sequences at random to which it was *not* reported to bind, to a *p*-value of 0.01 or less. These were used as the “negative” set. Orthologous sequence from *sensu stricto* species were included, aligned for PhyloGibbs-MP with Sigma version 1.1.3 (as in other benchmarks). In the case of PhyloGibbs-MP, predictions in the negative set were discarded (PhyloGibbs-MP does not distinguish between positive and negative sets), and only predictions from the positive set that arose from multiple tracked windows, at least one of which had a tracking score better than 0.2, were considered. The other programs reported at most one motif each for the positive set, and each such prediction was considered.

All input and output files, and detailed commandlines, are available at http://www.imsc.res.in/∼rsidd/phylogibbs-mp/supporting-data/.

### CRM Prediction

CRMs documented in the REDfly [33] database were chosen, that were under 3000 bp long. Surrounding sequence was included to bring the total length of the sequence to 10000 bp. If two CRMs lay within 15000 bp of each other, the associated sequence was fused into a single sequence. In this way, 234 CRM-containing sequences were identified in *D. melanogaster*. Orthologous sequence was selected from *D. pseudoobscura*, *D. yakuba*, and *D. simulans*. Orthology identification was made using multiple sequence alignments from VN Iyer, DA Pollard and MB Eisen (personal communication). The sequences were re-aligned with Sigma 1.1.3. PhyloGibbs-MP was run without priors and with priors constructed from the FlyReg database [38] (see below). Stubb (version 2.1) and Cluster-Buster were run using the FlyReg priors. Cis-Module requires no priors. EMC-Module requires a prior set of known binding sites, so the FlyReg priors were used to predict these sites (sites with a log-odds larger than 7 were included, and the specified width of sites was 8). PhyloGibbs-MP was run with 1, 2 (*melanogaster* and *yakuba*) or all 4 input sequences, aligned with Sigma 1.1.3; Stubb was run with 2 input sequences, *melanogaster* and *yakuba*. Other programs were run on melanogaster alone.

All input and output files, and detailed commandlines, are available at http://www.imsc.res.in/∼rsidd/phylogibbs-mp/supporting-data/.

### Construction of Prior Weight Matrices

The FlyReg [38] database of DNAse I footprints in *D. melanogaster* was used. Only those TFs were considered for which two or more footprints were available. For each footprint, orthologous sequence was extracted for *D. pseudoobscura*, *D. yakuba*, *D. simulans*, *D. erecta*, and *D. ananassae* using multiple sequence alignments from VN Iyer, DA Pollard and MB Eisen (personal communication). Thus, where *N* footprints may have been available in FlyReg, up to 6*N* sequences were used including orthologous sequences. The command-line for PhyloGibbs-MP was chosen via a heuristic depending on the number and lengths of footprints. The detailed commandlines and output files, and the generated weight matrices, are available at http://www.imsc.res.in/∼rsidd/phylogibbs-mp/supporting-data/.
